# Aligning sustainability assessment with responsible research and innovation: Towards a framework for Constructive Sustainability Assessment

**DOI:** 10.1016/j.spc.2019.05.002

**Published:** 2019-10

**Authors:** Nicholas E. Matthews, Laurence Stamford, Philip Shapira

**Affiliations:** a Manchester Institute of Innovation Research, Alliance Manchester Business School, The University of Manchester, Booth Street West, Manchester, M15 6PB, UK; b Manchester Synthetic Biology Research Centre for Fine and Speciality Chemicals, Manchester Institute of Biotechnology, The University of Manchester, 131 Princess Street, Manchester, M1 7DN, UK; c School of Chemical Engineering and Analytical Science, The University of Manchester, The Mill, Sackville Street, Manchester, M1 3AL, UK; d School of Public Policy, Georgia Institute of Technology, Atlanta, GA 30332-0345, USA

**Keywords:** Emerging technologies, Sustainable development, Sustainable production, Life-cycle assessment, Responsible research and innovation

## Abstract

Emerging technologies are increasingly promoted on the promise of tackling the grand challenge of sustainability. A range of assessment and governance approaches seek to evaluate these claims, but these tend to be applied disparately and lack widespread operationalisation. They also face specific challenges, such as high levels of uncertainty, when it comes to emerging technologies. Building and reflecting on both theory and practice, this article develops a framework for *Constructive Sustainability Assessment* (CSA) that enables the application of sustainability assessments to emerging technologies as part of a broader deliberative approach. In order to achieve this, we discuss and critique current approaches to analytical sustainability assessment and review deliberative social science governance frameworks. We then develop the conceptual basis of CSA - blending life-cycle thinking with principles of responsible research and innovation. This results in four design principles – transdisciplinarity, opening-up, exploring uncertainty and anticipation – that can be followed when applying sustainability assessments to emerging technologies. Finally, we discuss the practical implementation of the framework through a three-step process to (a) formulate the sustainability assessment in collaboration with stakeholders, (b) evaluate potential sustainability implications using methods such as anticipatory life-cycle assessment and (c) interpret and explore the results as part of a deliberative process. Through this, CSA facilitates a much-needed transdisciplinary response to enable the governance of emerging technologies towards sustainability. The framework will be of interest to scientists, engineers, and policy-makers working with emerging technologies that have sustainability as an explicit or implicit motivator.

## Highlights

• Analytical and deliberative approaches to assessing emerging technologies are reviewed and critiqued.
• A new conceptual framework is developed which blends complementary deliberative and analytical approaches.
• Four design-principles are proposed for evaluating the sustainability of emerging technologies.
• A concise but flexible three-step methodology is outlined to demonstrate how deliberative and analytical approaches can be aligned.

## Graphical abstract

**Figure:**
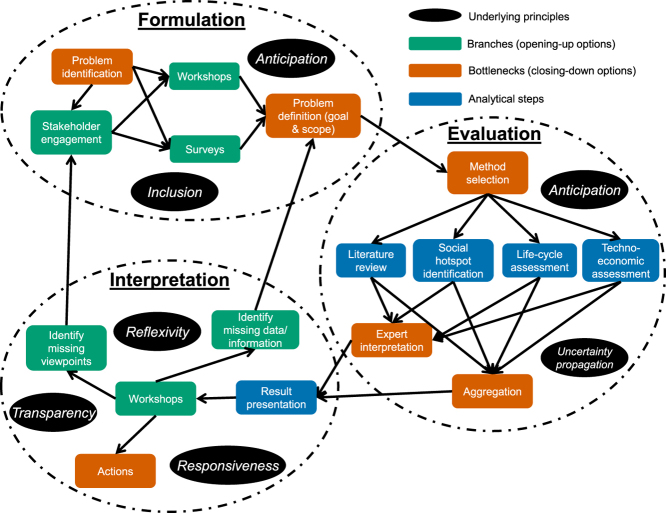

## 1 Introduction

While aggregate economic growth and urbanisation continue at a global level, conveying improved opportunities, health and quality of life for many, this is occurring at the expense of the environment with the costs and benefits of development unevenly distributed (UN, 2012). Recognition of these problems has led to the emergence of sustainable development as a dominant paradigm to mobilise governance and policy responses, defined by the 1987 Brundtland report as follows:

*“Sustainable development is development that meets the needs of the present without compromising the ability of future generations to meet their own needs”.* (Brundtland, 1987)

Through the United Nations (UN), multiple attempts have been made to bring about coordinated international efforts towards sustainable development. Of particular note are the 17 sustainable development goals (SDGs) which span a range of environmental, social and economic areas (UN, 2015).

With the pressing challenge of sustainability, there is a growing focus on how emerging technologies could provide potential solutions to sustainability challenges. Recent examples include synthetic biology, nanotechnology, and artificial intelligence. Disruptive scientific and technological developments are anticipated in these domains which can be viewed in terms of Kuhnian style “paradigm shifts” as well as Schumpeterian examples of “creative destruction” (Kuhn, 1970, Schumpeter, 1942). The field of sustainability transitions suggests that disruptive innovation is essential in order to displace existing socio-technological regimes but that the transition to more sustainable modes is as much a social as a technical challenge, requiring an understanding of issues such as lock-in and path-dependency that exert powerful exclusion effects on new entrants (Kates et al., 2001, Kemp et al., 1998, Markard et al., 2012). Furthermore, these new, diverse and disruptive technologies are united by the common motivation, or promise, of improved sustainability, yet it is widely acknowledged that they can yield both “good” and “bad” outputs and can create significant winners and losers (Balmer and Martin, 2008, van den Belt, 2013, Yuste et al., 2017). Thus, the sustainability promise associated with emerging technologies cannot be taken as an assumed fact; rather, critical evaluation is required, from both technical and social points of view, ideally at the early stages of development.

Recently, there has been a policy drive towards developing technologies that contribute to sustainable development, an example being the European Commission’s eco-innovation initiative (EC, 2013). At the same time, policy (reflecting public concerns) increasingly seeks for emerging technologies to be governed in a manner that is in line with societal priorities, encapsulated within the concept of responsible research and innovation (RRI) (EC, 2013; Stilgoe et al., 2013; SBRCG, 2012. Linking these two aspects is the aspiration for innovations and technological change to be directed towards tackling societal “grand challenges”, including the pressing need to transition to a more sustainable society (von Schomberg, 2013).

To align emerging technological fields towards desired societal and sustainability outcomes requires knowledge concerning the sustainability impacts of technologies to be made available at low technology readiness levels (TRLs) in research, proof of concept, and testing phases. The concurrent application of sustainability assessments to emerging technologies as they emerge could allow technological advances to be taken forward in a sustainable manner. However, in the early phases of technological development, the data is neither known nor available to carry out established environmental assessments of an innovation. Yet, by the time the technology has developed, such that this data is available, much developmental flexibility has been lost as lock-in and path-dependence sets in. This challenge is often referred to as the Collingridge dilemma (Collingridge, 1980). Furthermore, such assessments require clear underlying definitions of sustainability and sustainable development, as well as a grasp of what society wants and needs from emerging technologies. The latter is also particularly uncertain during the early stages of development.

While early technological development that is concurrent and iterative with sustainability assessment is inherently problematic, we suggest that there are ways to navigate through this complexity. Assessment approaches from a variety of fields have been developed with the aim of generating knowledge to guide emerging technologies. These originated as analytical and expert-based assessment routines but have increasingly been augmented with more qualitative, deliberative and participatory approaches to assessing and governing emerging technologies developed in the social sciences (Stirling et al., 2008). We argue that both the deliberative and analytical approaches are complementary and aim to deconstruct the distinctions between them to develop a conceptual framework for *Constructive Sustainability Assessment* (CSA). By “constructive”, as we will discuss in subsequent sections, we mean inclusive processes of dialogue, interaction, and consideration of diverse groups in technological design and deployment. CSA grounds the state-of-the-art in sustainability assessment within deliberative methods to allow for the more open and mutually beneficial evaluation and governance of emerging technologies.

We start by discussing both analytical sustainability assessments and deliberative governance approaches. We then explore their potential complementarity, developing four principles of CSA which can guide the application of sustainability assessments to emerging technologies. Finally, we outline a practical, three-step methodology to operationalise CSA.

## 2 Assessing sustainability

**Fig. 1:**
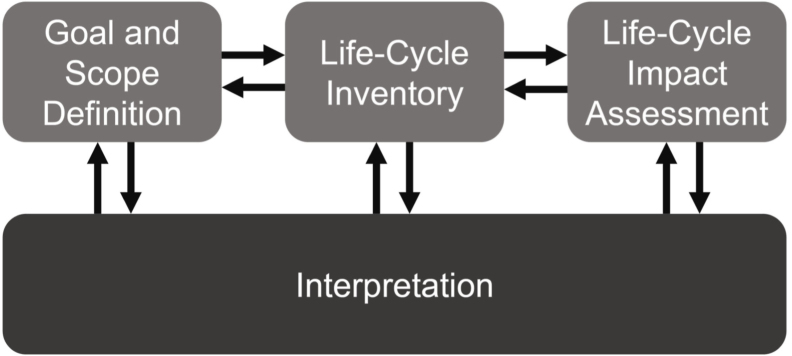
The ISO standards structure for an LCA (ISO, 2006a, ISO, 2006b).

Although numerous analytical approaches to assessing sustainability have emerged in recent decades (such as energy/exergy analysis and carbon/ecological footprinting), typically with a focus on environmental sustainability, the most widely applied and comprehensive methodology is that of life-cycle assessment (LCA) (Patterson et al., 2017). LCA was first developed in the 1970s, with a focus on reducing resource depletion and environmental damage (Klöpffer, 1997). A series of concerted efforts in the 1990s resulted in International Organisation for Standardisation (ISO) standards for LCA and the now widely familiar underlying structure for LCA studies shown in Fig. 1 (Guinée et al., 2011; ISO, 2006a, ISO, 2006b).

The LCA approach is underpinned by “life-cycle thinking” (LCT). LCT involves broadening the perspective when evaluating products and processes such that flows and impacts are considered throughout the life-cycle from “cradle-to-grave”^1^ (Azapagic, 2010). Taking a life-cycle perspective when evaluating sustainability seeks a holistic consideration of all possible impacts of a product, aiming to avoid temporal or geographic burden shifting and unexpected impacts. With the concern that the continued growth of production and consumption is pushing the earth to its biophysical limits, it is hoped that applying a life-cycle approach can help to achieve more sustainable patterns of consumption and production (Azapagic and Perdan, 2014).

Recently, LCA has undergone further infrastructural and methodological development. New approaches have emerged such as hybrid, economic input–output and anticipatory LCA (Wender et al., 2014a). There have also been developments in LCA databases (e.g. Ecoinvent, U.S. LCI Database) and software tools (e.g. openLCA, Brightway2) to open-up the application of LCA to a broader spectrum of practitioners (Finnveden et al., 2009). A number of life-cycle impact assessment (LCIA) methodologies have been proposed and improved upon (e.g. ReCiPe, CML), incorporating improved understanding of pollutant pathways, ecosystem, and human health impact mechanisms, as well as, in some cases, differing value systems. Attention has been raised to the issues of scale-up and its effect on LCA results with approaches put forward to better take into account scaling effects (Piccinno et al., 2016, Shibasaki et al., 2006; Simon et al., 2016). Using highly-aggregated datasets with limited primary data, screening level LCA studies have been deployed to allow hotspot identification during preliminary product development (Gasa and Weil, 2011, Upadhyayula et al., 2017). At the same time, the importance of handling and propagating uncertainty has been increasingly recognised, with sensitivity and uncertainty analysis forming key steps in many LCA studies (Finnveden et al., 2009, Gargalo et al., 2016).

## 3 Emerging challenges for analytical sustainability assessments

### 3.1 New responsibilities

Sustainability is progressively becoming an influential and crucial topic and an area of societal concern. As a result, sustainability assessments are increasingly employed by firms and governments who are applying life-cycle thinking to promote more sustainable decision-making (Sala et al., 2016, Sonnemann et al., 2018; UNEP/SETAC, 2008). Sala et al. (2015) conceptualise sustainability assessment as a tool for operationalising sustainability science and a systematic approach through which sustainable development goals can be achieved. This highlights an evolving view of what sustainability assessment can and should be mobilised to do, with an increasing focus on utilising analytical sustainability assessments to inform decision-making and governance to help facilitate transitions to a more sustainable society (Sala et al., 2015, Sonnemann et al., 2018).

Applying analytical assessments to emerging technologies is an area of particular promise as they represent technologies that have not yet been entrenched by path dependency and lock-in. Although there is debate about how emerging technologies can and should be defined (Rotolo et al., 2015), these are broadly technologies that are still “in-the-making” (Latour, 1987). This creates opportunities for assessment where knowledge or information that can be generated at the early stage of development has a greater potential to influence subsequent development and associated impacts. Early analytical assessments can be important in influencing the promises, commitments, and expectations of emerging fields which in turn shape the technological facts and objects that are created (Borup et al., 2006). Thus, the way in which early analytical sustainability assessments are carried out, framed, used and communicated becomes an even more crucial consideration and responsibility. To take up these roles and responsibilities, analytical sustainability assessments must address a number of challenges, as outlined below.

### 3.2 Broader notions of sustainability

Demonstrated by the wide remit of the UN’s SDGs, contemporary notions of sustainability span far beyond environmental considerations and biophysical limits to consider social and economic dimensions of sustainability (Azapagic and Perdan, 2014, Grunwald and Rösch, 2011; UN, 2015). To accommodate this, there has been a call within the sustainability assessment community to “broaden the scope” of LCA (Jeswani et al., 2010, Weidema, 2006). Life-cycle sustainability assessment (LCSA) tackles this by taking a three pillars approach to sustainability assessment, combining LCA with life-cycle costing (LCC) and social life-cycle assessment (SLCA) (Finkbeiner et al., 2010, Kloepffer, 2008, UNEP/SETAC, 2011; Zamagni et al., 2013).

However, of the three components of LCSA, neither LCC or SLCA have reached the level of method development or standardisation seen for LCA. LCC has long been applied alongside LCA (Norris, 2001) and while a code of practice has been developed to align it with LCA (Swarr et al., 2011), LCC lacks consensus over how it should be applied or, indeed, whether it is a relevant part of LCSA at all (Jørgensen et al., 2010; Wood and Hertwich, 2013). SLCA, on the other hand, represents a younger and less consistent concept. Guidelines were laid down by UNEP/SETAC almost 10 years ago with an update under development (UNEP/SETAC, 2009, UNEP/SETAC, 2013). Progress has also been made to address data shortages, for example with the development of the social-hotspots database and PSILCA (Benoit-Norris et al., 2012, Ciroth and Eisfeldt, 2016). However, while the literature surrounding SLCA is growing rapidly (Petti et al., 2016), the lack of standardisation remains a persistent issue (Arcese et al., 2016, Grubert, 2016, Kühnen and Hahn, 2017; Russo Garrido et al., 2016).

The inclusion of social impacts brings increased challenges to the field of analytical sustainability assessment. Assessing social aspects greatly increases levels of subjectivity and requires a move away from the positivist epistemology used in LCA and LCC. It is therefore increasingly agreed that if (S)LCA is going to robustly grapple with the social dimension within sustainability assessments then there is a need to embrace the role of the social sciences (Azapagic and Perdan, 2014, Grubert, 2016, Iofrida et al., 2018; Sala et al., 2013). Qualitative approaches applied in the social sciences can assess social impacts for which no quantitative metric can be fully or readily derived but this is epistemologically inconsistent with traditional environmental LCA which applies a positivist worldview reflecting the engineering paradigms it developed within (Iofrida et al., 2018). At the early stages of technological development, the assessment of environmental and economic aspects may also benefit from employing these more qualitative approaches given the high levels of uncertainty. As a result, future sustainability assessments will necessitate a multi-paradigm approach to marry different epistemological positions under a single framework (Lang et al., 2012). This represents a fundamental challenge.

### 3.3 The limitations of analytical approaches

Sustainability assessment methodologies, such as LCA, might provide a means through which the sustainability promises of emerging technologies can be evaluated and unexpected impacts identified at low-TRLs such that certain technological trajectories can be avoided or impacts mitigated. However, typically, LCA approaches rely on detailed and specific data from throughout the life-cycle of processes and products that are already in the market (Cherubini et al., 2009, Spath et al., 1999, Vink et al., 2003; Williams et al., 2006). In recent years an increasing volume of research has attempted to conduct LCA on products at low TRLs, such as nanomaterial production, graphene, biofuels and carbon capture and utilisation (Arvidsson et al., 2014, Cuellar-Franca et al., 2016, Gavankar et al., 2015, Hischier and Walser, 2012, Rajagopalan et al., 2017, Wender et al., 2014b). While it appears that the existing underlying framework for conducting LCA is appropriate for emerging technologies, there is no well-established approach to the use of LCA under such circumstances (ISO, 2006a, ISO, 2006b, Klöpffer et al., 2007). Furthermore, these analytical approaches face a number of limitations:

• No assessment can ever be fully objective. LCA studies involve subjective judgements relating to system boundaries, data sources, allocation, impact assessment, and aggregation. Subjective decisions, assumptions, and limitations are an inherent feature of any modelling approach, particularly when new methods are being developed. While the ISO standards for LCA provide guidance for transparency by clearly setting out the “goal and scope” of any assessment (ISO, 2006b), a great deal of “black-boxing” still occurs, and there is little guidance on how these subjective decisions should be made.
• Using Monte Carlo simulations and probabilistic comparisons can enable the high parameter uncertainty experienced at low-TRLs to be propagated and explored (Wender et al., 2014b). However, this uncertainty is not always communicated in the results, with many studies still attempting to present simple, aggregated results, which can be misleading for policy-makers (Stirling, 2008). Moreover, the ISO standards for LCA do not explicitly detail the need, method or communication of any formal uncertainty analysis.
• It is inevitable, particularly for emerging technologies, that some aspects will not be responsibly measurable: at some threshold of statistical uncertainty, the existing analytical methods for handling uncertainty must surely become insufficient (Hetherington et al., 2014). However, while a quantity or aspect being unknown or unmeasurable does not make it any less significant, a purely analytical assessment would simply omit it as a known unknown.
• Unknown unknowns are prevalent when assessing emerging technologies with limited available data and knowledge. Previous technologies promoted on the grounds of (environmental) sustainability have proved to have questionable sustainability performance once further information comes to light: a notable example being first generation biofuels and bio-based plastics when indirect land-use change is taken into account (Piemonte and Gironi, 2011, Searchinger et al., 2008).
• An unavoidable constraint is that of limited resources, in terms of skills, time and/or money. The application of complex analyses during periods of rapid technological development will inherently involve tough choices with respect to the allocation of resources, potentially leading to incomplete assessments (Peace et al., 2017).

A final caveat is more fundamental. Definitions of sustainable development hinge around concepts of intergenerational equity and of maintaining quality of life now and in the future by working within our biophysical limits. However, while these biophysical limits represent phenomena which can be measured against, the kind of world and society that should be maintained within those limits involves subjective and value-laden judgements (de Vries and Petersen, 2009) Furthermore, the role and relevance of sustainability assessment tools in informing the broader field of sustainability science and guiding the path to sustainable development is essentially dependent on the worldviews and values of those individuals and stakeholders concerned (Asveld and Stemerding, 2016). Depending on differing views of knowledge and of nature, the ways in which sustainability assessments would be interpreted, or indeed whether they are relevant at all, may differ markedly (Asveld et al., 2014, de Vries and Petersen, 2009; Hofstetter et al., 2000). Thus, analytical approaches on their own will never be sufficient to fully address societal concerns surrounding emerging technologies.

These critiques should not be construed as a critique of analytical sustainability assessments themselves, which frequently yield valuable and robust quantitative findings that can inform sustainability minded governance and decision-making. On the contrary, the field of analytical sustainability assessment has progressed and developed considerably in response to the new challenges it faces as it is increasingly tasked with helping to deliver a more sustainable society. Rather, the problem lies in the way in which analytical approaches are employed and communicated. All too often, analytical approaches alone like LCA are asked to answer specific sustainability questions yet when it comes to the concept of sustainability and subjective decision-making, there is only so much that an analytical perspective can inform. In all cases, but particularly for emerging technologies, employing purely analytical approaches yields a substantially incomplete picture.

To address these challenges, we suggest that the field of analytical sustainability assessment must continue to evolve and progress, operationalising a more transdisciplinary approach, engaging in active dialogue with stakeholders to position sustainability assessments within broader societal contexts, and considering how sustainability assessments can be pragmatically applied to explore rather than answer sustainability questions and communicate this within sometimes-restrictive industry and policy contexts. Such changes are already well underway and will result in a methodology almost unrecognisable from the early roots of LCA. Sustainability assessments must continue to expand from their analytical roots, and draw upon the experiences and approaches of other fields, particularly those that deal with the challenges of assessing and governing emerging technologies, such as technology assessment and deliberative governance frameworks.

## 4 Technology assessment and deliberative governance

### 4.1 From technology assessment to responsible innovation

The formal elaboration of technology assessment (TA) arose in the latter half of the 20th Century, reflecting an aim of “reducing the costs of trial and error learning” by anticipating potential social and technical problems associated with emerging technologies (Schot and Rip, 1996). A key point in this movement was the establishment in 1972 of the Office of Technology Assessment (OTA) by the US Congress, with further TA offices formed in Europe such as the Netherlands Office for Technology Assessment (NOTA, now the Rathenau Institute). The emergence of TA reflected, explicitly or implicitly, an anticipation of what is now known as the Collingridge dilemma (noting that the emergence of TA precedes Collingridge’s 1980 book) (Nordmann, 2010).

While early forms of TA were critiqued as too expert based (van Lente et al., 2017), there was early recognition of the “heavily entangled” nature of technology and society and therefore that assessments can never be truly objective or value-free (Bijker et al., 1987, Lee and Bereano, 1981). Subsequently, there were efforts to engage with broader stakeholders to facilitate the co-production of emerging technologies. An early example was constructive technology assessment (CTA), pioneered by NOTA in the 1980s–1990s (Schot and Rip, 1996). CTA aims to inform decision making surrounding technologies by anticipating impacts while taking a constructive approach, where “design criteria” for technologies are developed in an open and inclusive process, helping to facilitate societal alignment of emerging technologies (Ribeiro et al., 2018, Schot and Rip, 1996). CTA focusses on bridging gaps between technological actors and wider society by facilitating interactive workshops and other “bridging events” which can provide spaces for anticipation and reflexivity (Rip, 2018). These events may help to reduce and actively manage uncertainties surrounding impacts and societal responses.

The development of CTA marked a key shift in focus away from government or parliament centred approaches of TA orientated towards informing policy, towards more distributed approaches (Lee and Bereano, 1981). CTA laid the groundwork for subsequent developments such as real-time technology assessment (RTTA), anticipatory governance (AG), and responsible research and innovation (RRI) (Guston, 2014, Guston and Sarewitz, 2002, Stilgoe et al., 2013). Such approaches are characterised by a closer relation to the development of technology itself, emphasising distributed responsibility for technological development amongst promoters and enactors. These changes reflect an increasing recognition of the potential problems, as well as benefits, created by emerging technologies alongside a fundamental reframing of technological innovation away from the view of research and development as an intrinsic public good (van Est, 2017). They therefore exist in a new context, one where there is a wish, or rather perhaps an urgent need, to actively shape future socio-technological systems towards desired societal and sustainability goals (Fleischer and Grunwald, 2008, Kemp et al., 1998, Nordmann, 2010). For convenience, we shall refer to this family of approaches as “deliberative approaches”, recognising their common emphasis on multi-stakeholder deliberation and goal of opening-up discussions around emerging technologies (van Lente et al., 2017).

Reflecting a growing public policy drive for emerging technologies to yield broadly-distributed “public goods” as well as tackle “grand challenges”, attempts have been made more recently to further institutionalise deliberative governance approaches within technological R&D projects to foster responsible innovation (Willetts, 2013, Roco et al., 2011; von Schomberg, 2011). In the United States, two social science research centres were incorporated within the National Science Foundation’s nanoscale science and engineering research programme leading to the development of real-time technology assessment and anticipatory governance approaches that attempt to tackle the Collingridge dilemma by leaving “…that relationship between governing decision and quality of knowledge in productive tension” (Guston, 2014). An aim was to provide instruments to enable the co-construction of emerging technologies towards societal needs using widespread public engagement, participatory scenario development and integration of social and natural scientists within research environments, distributing responsibility throughout a variety of actors in technological development (Guston, 2014).

Meanwhile, in Europe, RRI has been incorporated into research programmes at both the European and national levels (Clarke and Kitney, 2016; EC, 2017). One influential framework advocates embedding principles of anticipation, inclusion, reflexivity, and responsiveness into the research and innovation process (Stilgoe et al., 2013). The core elements of this framework were adopted by the UK’s EPSRC (2019). Inclusion reflects the increasingly recognised need to engage relevant stakeholders early, to ensure the appropriate social values are considered in technology development (Delgado et al., 2011, Wilsdon and Willis, 2004). Responsiveness emphasises the importance of being able to modulate trajectories as knowledge of impacts and stakeholder values develops. A critique of precursors to RRI was the lack of institutionalised responsiveness, performing more observatory roles instead (Zwart et al., 2014). Reflexivity refers to a level of self-awareness within the institutions, governance structures and actors which are involved in scientific developments, and involves being open-minded to one’s own assumptions and framings (Stilgoe et al., 2013). Finally, anticipation is a process of “capacity building” through the generation of technology visions and imaginaries, drawing from anticipatory governance (Guston, 2014).

### 4.2 Limitations and challenges of deliberative approaches

While deliberative approaches have flourished conceptually, challenges in operationalisation persist. CTA is described as having a “diffuse and emerging character” while RRI has been described as a “mobilising concept” (Ribeiro et al., 2016, Schot and Rip, 1996). Such frameworks may be highly useful to bring together and mobilise interdisciplinary academic perspectives, but as a result there is also a distinct lack of clear and practical methodological guidance. Another issue has been that funding for social science research into specific emerging technologies has historically followed several years after the initial natural science funding commitments and on a much smaller scale, limiting the scope for assessments to be carried out and alternative perspectives included (Guston, 2014).

Furthermore, while public engagement is a fundamental feature of the deliberative governance approaches we have described, the role of public participation in assessment processes is still one of contestation. While much of the recent literature, coupled with policy commitments along the same lines, emphasises the need for public participation in science and technology such as through engagement with assessment and appraisal activities, there is little agreement on when and how this should take place (Delgado et al., 2011, Stilgoe et al., 2014). “Upstream engagement” activities can be particularly challenging in terms of identifying relevant stakeholders and implementing appropriate participatory activities while avoiding artificial framing and closing-down of discussions (Brandt et al., 2013; Lang et al., 2012, Wilsdon and Willis, 2004).

The move towards more participatory and deliberative methods for assessing and governing emerging technologies, as part of a more open-ended and democratic approach, could be seen as an alternative to analytical, expert-led approaches. However, particularly in the case of emerging technologies, there are similar risks relating to normative framing of the engagement activities which could bias the outcomes, potentially towards instrumental goals. In the case of nanotechnology, it has been claimed that engagement tended to “close down” discussion and failed to reject the linear, determinist view of technological “progress” (Delgado et al., 2011).

Participatory approaches are pitched as democratising technological development as a means to achieve “societal alignment” (Ribeiro et al., 2018). However, others suggest that the idea of representing all members of “society” is highly problematic, and practically impossible (van Lente et al., 2017). Furthermore, sustainable development is underpinned by a consideration of the wants and needs of future generations alongside those in the present (Brundtland, 1987). Therefore, employing participatory approaches to guide sustainable technological development may risk prioritising present generations over future generations who cannot represent themselves.

These criticisms notwithstanding, we do not argue that increased participation of publics and the use of deliberative approaches in technological development processes are unimportant or ineffective. Indeed, going forward such approaches are vital and need to be enhanced to better align technological development with societal goals and needs. The capacity of public engagement to include marginalised voices and contribute to the co-construction of technology should be welcomed. Still, the use of public engagement does not preclude many of the issues of power, representation, legitimation, and framing that pervade analytical approaches (Stirling et al., 2008). As Stirling et al. (2008) articulate, the important distinction is not between participatory and analytical approaches, but between opening-up and closing-down of technological options, with current approaches tending to close-down. Insincere, narrowly framed, and poorly executed engagement is more of a risk than no engagement at all, as it grants the assessment the perceived trust and legitimation of a participatory approach. If improperly used, engagement can act as a smoke screen, and risks being used for instrumental purposes.

More practically, while deliberative governance approaches have demonstrated the utility of participation and deliberation in order to integrate alternative forms and frames of knowledge into early technological development, they pay little attention to the complementary role that more expert-based analytical assessments might play. According to Grunwald (2010), the combination of explanatory knowledge (such as from analytical assessment) with orientation knowledge (such as derived from participatory approaches) is essential to enable informed, action-guided knowledge production to help achieve sustainable development. Thus, while the original rejection of purely expert-based assessment approaches may have been well-justified, a more nuanced view of their potential complementarity is required in order to generate the necessary interdisciplinary knowledge to guide the sustainable development of emerging technologies. We seek to improve the quality and social utility of RRI and related governance approaches, moving away from viewing RRI and other deliberative approaches as substitutes for analytical sustainability assessments (and vice-versa), and towards exploring how they can complement one another.

## 5 Towards a constructive sustainability assessment

For emerging technological developments to be taken forward in a sustainable manner, and to deliver on the promises they are promoted upon, there is a need to evaluate the sustainability of technologies as they emerge, requiring the management and tackling of issues relating to uncertainty conceptualised within the Collingridge dilemma. We have so far made the case that neither participatory nor analytical approaches to assessing and governing emerging technologies towards sustainability are in themselves sufficient to do this. Available approaches to do so tend to close-down discussions and can lead to narrow framing of the sustainability concepts, questions, and priorities.

We emphasise the complementarity between the analytical and deliberative approaches discussed. Indeed, in order to fully grapple with the inherently subjective and value-laden concept of sustainability, to assess social impacts, and to introduce participatory methods to help relate sustainability assessment outputs to their broader societal context, there has been a clear and repeated call for greater inclusion of social science methods, theories and perspectives within sustainability assessment frameworks like LCA as part of a transdisciplinary approach to sustainability science (Azapagic and Perdan, 2014, Iofrida et al., 2018, Sala et al., 2013, Thabrew et al., 2009).

While participatory approaches have thus-far evolved along separate streams from analytical sustainability assessment, there now exist the necessary drivers to facilitate productive cross-fertilisation. The call from many authors to embrace the social sciences’ role in sustainability assessment is gaining momentum, while the growing number of fields and disciplines attempting to tackle sustainability issues provides the necessary academic groundings to tackle the many dimensions that make up the complex challenge of sustainable development. Furthermore, although similar spaces still need to be established more widely in industry (Flipse et al., 2014), the institutionalisation of social science research within emerging technology research programmes such as those relating to synthetic biology and nanotechnology provides spaces to facilitate the necessary interdisciplinary research (Balmer et al., 2016). Within these spaces, alignment with analytical assessments could act as a route in for more deliberative approaches to engage with and influence the trajectories of emerging technologies. Talking about data and quantitative models, such as can be generated from LCAs, helps social scientists to better understand the language of natural scientists and therefore makes the research and knowledge that is subsequently (co-)produced relevant, understandable and persuasive.

Building on a small but burgeoning literature, we now aim to blend these different approaches into a coherent framework for *Constructive Sustainability Assessment* (CSA) through which the necessary interdisciplinary assessments can be operationalised. CSA builds on theoretical underpinnings and frameworks from both the social and natural sciences (see Table 1). Conceptually, we draw mostly on frameworks for deliberative governance situating CSA close to technological development (Table 2), emphasising the importance of deliberative and discursive approaches informed by explanatory knowledge generated through analytical assessments to enable informed and incremental decision making under uncertainty (Grunwald, 2010). CSA can facilitate the exploration of sociotechnical scenarios in interactive workshops to “enhance reflexivity through anticipation and learning” (Schot and Rip, 1996). Grounding analytical assessments within deliberative governance can thus help to achieve the “reflexive sustainability assessment” called for by Fleischer and Grunwald (2008). This enables an iterative process of informed experimentation which provides crucial opportunities for learning in support of sustainability transitions (Luederitz et al., 2017).

We now articulate the CSA framework through four core design principles which capture the complementarity between analytical sustainability assessments and participatory and deliberative approaches, distilling the conceptual links between the two areas of study. This gives a theoretical underpinning to CSA which is grounded in both the social and natural sciences.

### 5.1 Design principle 1: Transdisciplinary approach

The sustainability challenges faced by society (e.g. climate change, water and food scarcity, equitable economic development) fundamentally span social and natural domains (Kates et al., 2001; UN, 2015). Analytical sustainability assessment can help to evaluate emerging technologies in relation to the biophysical limits of the earth, in terms of ecosystem and human health as well as resource scarcity. However, even given knowledge of their impacts, the governance of emerging technologies to maximise well-being is a question that requires a societal response (de Vries and Petersen, 2009). Therefore, CSA requires the integration of analytical knowledge with knowledge of sustainability goals and criteria (Grunwald, 2010).

In attempting to marry social science and natural science theories and practices, engaging in deliberative activities alongside analytical assessments, CSA builds on the fact that research is increasingly undertaken as part of multi- or trans-disciplinary teams (Gibbons et al., 1994, Leydesdorff and Etzkowitz, 1996). Applying these transdisciplinary approaches, where knowledge is co-produced through an interactive and integrated approach across numerous actors is a challenge and requires grappling with the differing backgrounds, academic vocabulary, methods and epistemological positions held by the researchers and societal actors involved.

### 5.2 Design principle 2: Opening-up perspectives

A challenge to both analytical and deliberative approaches is that they tend to close-down discussion and promote a linear view of technological development. If emerging technologies are to be aligned to societal needs, it is essential to integrate a wider range of perspectives in the assessment process and in the formation of expectations which shape future technological

developments (Borup et al., 2006). A way to initiate this is for assessments to actively engage with the viewpoints of a wide range of stakeholders. This requires sustainability assessment practitioners to move out of their “ivory towers” and engage with societal actors (Wiek et al., 2012).

**Table 1:** A comparison of selected technology assessment and governance routines with CSA.

Aspect	Traditional LCA	LCSA	RRI, CTA and anticipatory governance	Anticipatory LCA	Solution focussed sustainability assessment	Constructive sustainability assessment
*Discipline*	Natural sciences	Natural sciences (mostly)	Social sciences	Interdisciplinary (but mostly natural sciences)	Interdisciplinary	Interdisciplinary
*World-view*	Typically follows a hierarchist, “controlled nature” worldview	Typically follows a hierarchist, “controlled nature” worldview	Can handle differing worldviews	Typically follows a hierarchist, “controlled nature” worldview	Can handle differing worldviews	Can handle differing worldviews
*Perspective*	Retrospective	Mixed	Anticipatory	Anticipatory	Solution-focussed	Anticipatory
*Handling of uncertainty*	Largely ignored	Increasingly acknowledged and reported	Embraced and acknowledged	Embraced, propagated and rationalised	Unclear	Embraced, propagated and rationalised
*Opening-up or closing-down options?*	Closing-down	Closing-down	Opening-up (in theory)	Closing-down	Closing-down	Closing-down and opening-up
*Sustainability aspects*	Environmental focus	Can span environmental, economic and social aspects	Typically focusses on social aspects of emerging technologies	Environmental focus	Can span environmental, economic and social aspects	Can span environmental, economic and social aspects
*Sustainability definition*	Assumed/prescribed	Assumed/prescribed	Open	Assumed/prescribed	Determined through deliberation	Determined through deliberation
*Standardisation*	Established (ISO 14040/14044)	Increasing (e.g. SLCA guidelines)	Some (e.g. AIRR and AREA frameworks)	In development	Seven step approach	Standard approach at a high-level, flexible application

CSA is fundamentally stakeholder focussed, not least because the users of the assessment (e.g. decision-makers in the public and private sectors) are considered integral stakeholders. CSA requires the inclusion of a wider range of perspectives and values in the assessment process than typical analytical assessments allow, maintaining an open discussion of possibilities and interpretations. Engaging with a range of stakeholders is one approach to open-up discussions but, as has previously been highlighted, any attempt at widespread societal engagement activities must be done thoroughly and comprehensively, a challenge that will be further explored later.

### 5.3 Design principle 3: Exploring and communicating uncertainty

Assessing sustainability at low TRLs involves dealing with inevitable data gaps, normative ambiguities and unknown unknowns which are unresolvable at such an early stage of development (Hetherington et al., 2014, Olsen et al., 2018; van de Poel et al., 2017). Inspired by a recently developed anticipatory LCA (A-LCA) approach, CSA takes a prospective and anticipatory approach to sustainability assessment, embracing uncertainty as a fundamental feature of the assessment (Wender et al., 2014a, Wender et al., 2014b).

**Table 2:** A review of selected deliberative governance frameworks and their conceptual contribution to CSA.

Framework	Definition	Novelty	Core principles	Contribution to CSA
*Constructive technology assessment*	“A notion of shared responsibilities for managing technology in society, with all actors working toward the CTA goals of learning, reflexivity, and anticipation.” (Schot and Rip, 1996)	Inclusion of a broad range of actors in the design of technologies.	• Reflexivity • Co-production • Modulation and learning • Anticipation.	• The use of bridging events. • Inclusion of a broader range of perspectives.
*Anticipatory governance*	“A broad-based capacity extended through society that can act on a variety of inputs to manage emerging knowledge-based technologies while such management is still possible” (Guston, 2014)	Closer link to the process of technological development.	• Anticipation • Foresight • Engagement • Integration	• Integration of natural and social sciences. • Taking an incremental approach to governance.
*Responsible research and innovation*	“Taking care of the future through collective stewardship of science and innovation in the present” (Stilgoe et al., 2013)	Greater attention to normativity. Innovation to tackle grand challenges.	• Anticipation • Reflexivity • Inclusion • Responsiveness	• Directing innovation towards “normative anchor points” (von Schomberg, 2011) • Importance of embedding reflexivity and maintaining responsiveness.

Crucially, while A-LCA focusses on exploring issues of statistical uncertainty using Monte Carlo simulations and probabilistic comparisons, this tackles only one of the many sources of uncertainty (list adapted from (van de Poel et al., 2017):

• Parameter and scenario uncertainty;
• Uncertainty surrounding unknowns (both known and unknown) and rebound effects; and
• The subjectivity inherent in relation to societal priorities, values, and worldviews.

CSA extends anticipatory LCA to consider and embrace non-statistical uncertainties through participatory exploration of scenarios, alternative viewpoints, and unknown unknowns. Crucially, CSA acknowledges that uncertainty is a fundamental feature of assessing, evaluating, and governing emerging technologies, but also asserts that within the available uncertain data and information there is a great deal of knowledge that can be extracted to inform decision making. Indeed, rather than see uncertainty as a limitation it should be viewed as an opportunity. High levels of uncertainty, as well as reflecting our limited knowledge, also reflects the existence of flexibility and open design options to be explored (Grunwald, 2010). However, it is imperative when handling uncertainties that they are propagated throughout the process and that those using results are aware of their limitations. Uncertainties, unknowns, and unmeasurables must be communicated so as not to give misleading certainty which could result in uninformed and detrimental governance (Stirling, 2010).

### 5.4 Design principle 4: Anticipation of futures (not predicting solutions)

In focussing on the assessment and governance of emerging technologies, CSA takes a forward-looking, anticipatory approach to sustainability assessment. Through anticipating and reflecting upon future impacts, CSA facilitates capacity building such that organisations and individuals are better prepared for future challenges and developments, improving responsiveness (Guston, 2014). This then allows for the Collingridge dilemma to be actively managed, ensuring that as new information becomes available, technological actors are well equipped to respond rapidly (Stilgoe et al., 2013).

With the broad scope of platform emerging technologies like synthetic biology and nanotechnology, life-cycle tools offer opportunities for the exploration of the specifics and complexities of individual applications (Ribeiro and Shapira, 2019). Thorstensen and Forsberg (2016) articulate SLCA as a tool for anticipation at the level of specific products, allowing the systematic study of social sustainability issues, and operationalisation of RRI. According to Wender et al. (2014b), life-cycle tools enable an approach which: “systematically and iteratively explores uncertainties across the life cycle of an emerging technology to prioritise research with the greatest potential for environmental improvement and contributions to responsible innovation*”.* Helping technological actors to view and understand the variety of avenues and possibilities available and their wide-ranging implications helps to open-up governance approaches, questioning current technological expectations and commitments and promotes governance that emphasises informed experimentation and “directed incrementalism”, preserving developmental flexibility for longer (Grunwald, 2010).

## 6 Constructive sustainability assessment in practice

A step-wise approach to carry-out stakeholder grounded sustainability assessments has previously been outlined by Zijp et al. (2016) in the form of Solution-focussed Sustainability Assessment (SfSA). By blending state-of-the-art modelling alongside deliberative methods such as workshops and qualitative evaluation as part of a transparent and participatory sustainability assessment, SfSA utilises sustainability assessment to explore solutions to supposedly “wicked” sustainability problems (Zijp et al., 2016). CSA has a similar structure, but while SfSA starts with a sustainability problem and searches for solutions, CSA starts with emerging technologies and probes the promises of sustainability they are promoted upon. Thus, CSA aims to open-up discourse and explore options, rather than explicitly search for solutions.

A typical application of CSA would be within or by an organisation, and in this CSA has strong similarities to life-cycle management (LCM), which aims to provide a toolkit for organisations to integrate sustainability into management decisions (Hunkeler et al., 2003). However, LCM provides little guidance on the practicalities of carrying out sustainability assessments within organisations, remaining largely conceptual (Bey, 2018). While CSA does provide practical guidance, it is also not restricted to organisational contexts, and could easily be applied at higher levels, for example, to evaluate and inform national or international policies.

**Fig. 2:**
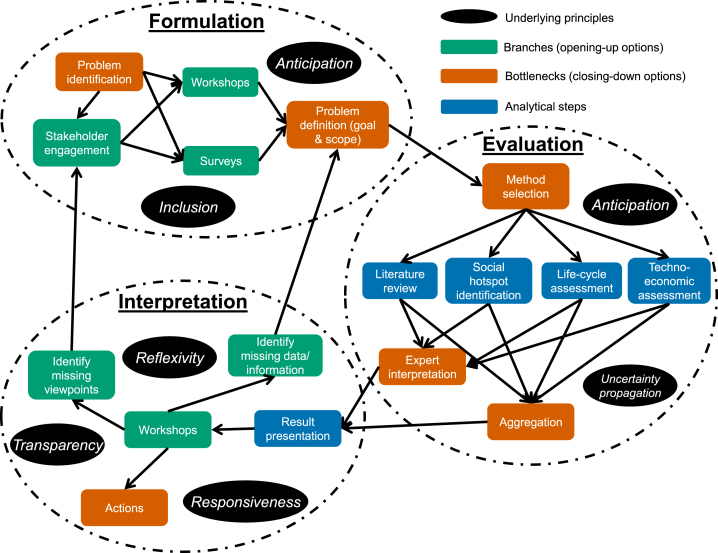
Methodological approach to CSA. Source: Authors’ elaboration.

This section refers to the assumed role of the “CSA practitioner(s)” who would facilitate the CSA process. A three-step process (Fig. 2) is deployed to operationalise the design principles outlined in the previous section. This begins with the *formulation* of the sustainability problem, which informs and guides the subsequent *evaluation* process, where the sustainability of the technology/product is assessed relative to the sustainability concept and priorities identified during problem formulation. Finally, the *interpretation* stage involves deliberative reflection and discussion of the results to identify outcomes, actions and, priorities for further study.

It is intended that CSA should be carried out in a cyclic or iterative manner to allow continuous constructive assessment and an incremental and adaptive governance approach (a view of public policy practice highlighted by (Lindblom, 1959), but refined in recent years as an explicit method, see (Andrews et al., 2015, Kuhlmann et al., 2019). Fig. 2 represents this diagrammatically. Stages such as method selection and data interpretation are key steps of the assessment process in that they allow possibilities to be explored and promises to be probed but also tend to lead to “closing-down” of options (represented by red/orange boxes in Fig. 2). Combining them with more open-ended methods such as workshops allows the process to be “re-opened” and alternative viewpoints “re-considered” (represented by green boxes in Fig. 2). Thus, the cyclic and continuous nature of the process is essential, not only to allow inclusion of new knowledge which is likely to improve over time and allow incremental governance but also to counter the tendency of evaluations and assessments to lead to gradual closing-down. The following sections articulate the three CSA steps in more detail. A “methodological toolkit” is provided in Table 3.

**Table 3:** A suggested toolkit for CSA.

Stage	Mobilised concepts, principles and frameworks	Methodological toolkit
Formulation	Co-construction; inclusion; ISO goal & scope definition	Stakeholder mapping; literature review; interviews; surveys; workshops; focus-groups
Evaluation	Anticipation; inclusion; ISO life-cycle inventory; ISO life-cycle impact assessment	Life-cycle assessment; social life-cycle assessment; Life-cycle costing; EIO modelling; hybrid LCA; screening LCA; up-scaling LCA; expert consultation; literature review; early-stage metrics; surveys
Interpretation	Value-sensitive design; reflexivity; responsiveness	Workshops; focus-groups; interviews; surveys; consensus conferences; citizen juries

### 6.1 Step 1: Formulation

#### 6.1.1 Stakeholder identification and engagement

Despite the requirement in the ISO LCA standards that the application, aims, audience, context, use and technical scope of the study are clearly defined in the “Goal and Scope Definition” stage, there is little guidance as to how this should be determined and no mention of stakeholders (ISO, 2006b). If these subjective judgements are made solely by LCA/CSA practitioners, the subsequent assessment will be framed relative to the sustainability visions and values of the practitioner (Freidberg, 2018). We propose that in a CSA process, the goal and scope definition phase is precluded by deliberative activities with stakeholders through workshops and surveys. This ensures that the subsequent sustainability assessment can be framed more broadly and inclusively as well as being made more explicit.

Mathe (2014) considers there to be four different kinds of stakeholders relevant for sustainability assessments: method users; result users; those affected by the impacts (beneficially or detrimentally) and those with input into methodological issues. Taking Mathe’s classification, CSA practitioners should consider themselves stakeholders, as both method users and potentially methodology developers. This is in line with a more constructivist view of the role of the researcher. Other stakeholders involved should include result users, and potentially, impacted groups. In recent years a growing number of stakeholder and public dialogues have taken place surrounding emerging technologies, particularly synthetic biology, the results of which can allow the inclusion of a greater variety of stakeholder viewpoints (Bhattachary et al., 2010, Castell et al., 2014, Schmidt et al., 2009, Stilgoe and Kearnes, 2007) in addition to new initiatives for engagement and dialogue. Stakeholder engagement can also be broadened over time as societal interest increases or resources become available.

#### 6.1.2 Deliberation

It is important that sustainability assessments are not framed in terms of “what we can measure” and instead start with “what matters”. Then, one can analyse what can be reasonably analysed and provide transparency about what cannot be reasonably measured or evaluated at the time. To achieve this, CSA advocates undertaking deliberative activities which allow the sustainability concept employed to be discussed and clearly specified (Zijp et al., 2016). This also provides a space to reflect on what the stakeholders would like to achieve from the process. Engaging stakeholders who will use the outputs of the study at this early stage can advance their understanding of the sustainability assessment process, improving engagement and trust as well as building capacity. We suggest the following aspects to be considered at this stage:

• Identification of potential technological futures and scenarios of interest.
• Clarification of the object, level and system boundaries of analysis.
• Discussion of the sustainability concept. The UN SDGs may provide a “normative anchor point” for this discussion (UN, 2015; (von Schomberg, 2011).
• Exploration of the worldviews of the stakeholders, in particular how they perceive nature (Asveld et al., 2014, Hofstetter et al., 2000).
• Discussion of data sources as well as the interpretation and presentation of outputs to ensure that the subsequent results are understandable, useful and seen as legitimate by stakeholders (von Geibler et al., 2006, Zijp et al., 2016).

The answers to these normative aspects of the sustainability assessment will vary in each application of the CSA framework, and the validity and utility of the problem formulation that results will be inherently dependent on the stakeholder perspectives included. Carrying out this process and outlining the assumptions and subjective elements that underpin the study improves transparency and legitimacy.

### 6.2 Step 2: Evaluation

#### 6.2.1 Method selection and the place of life-cycle methods

The effectiveness of the CSA process is underpinned by a period of evidence collection where the sustainability implications of the object of analysis are assessed. The formulation stage provides guidance to CSA practitioners in undertaking this evidence collection. However, picking appropriate methods from the many available remains a key challenge and an area where closing-down might occur, particularly when resources are limited and the use of streamlined methods such as scanning LCA might be required (Peace et al., 2017, Wangel, 2018). The use of such approaches transparently reflected upon and communicated alongside the results, acknowledging any limitations.

Previous studies have highlighted the need for a “case by case” approach to evaluating emerging technologies, advocating situated and context-specific evaluation (Ribeiro and Shapira, 2019). Thus, the CSA framework does not prescribe specific methods or the way in which they should be applied, particularly considering that the perceived utility and validity of different approaches will depend on stakeholder worldviews (Asveld and Stemerding, 2016). However, the methods used should be consistent with the overarching principles of CSA and make appropriate use of previous methodological developments and state-of-the-art.

In most cases, particularly when applying CSA to specific products, it is anticipated that life-cycle tools will fulfil this evidence gathering role. LCSA provides a useful methodological framework to follow, as it allows the consideration of all three pillars of sustainability (Finkbeiner et al., 2010). Furthermore, application of life-cycle tools such as LCA and SLCA at low-TRLs provide a means through which uncertainties can be rationalised and future impacts anticipated and explored (Thorstensen and Forsberg, 2016, Wender et al., 2014a). For particularly early-stage studies there are various challenges, especially surrounding process scale-up, and a number of alternative approaches are available, as discussed by Broeren et al. (2017). Such challenges introduce uncertainty, which is discussed below.

#### 6.2.2 Handling uncertainty

A central challenge is how uncertainty is handled and propagated. Uncertainties concerning data and knowledge should be duly acknowledged and propagated into the empirical evaluation while inevitable assumptions, exclusions, and limitations should be systematically recorded for presentation alongside the results. Existing LCA tools already possess methods for this and many databases include qualitative or quantitative uncertainty scores (Ciroth and Eisfeldt, 2016). To handle parameter uncertainty Monte Carlo simulations have been utilised in a number of recent studies (Baral et al., 2018, Gargalo et al., 2016, Pérez-López et al., 2018). This allows uncertainties relating to input parameters to be propagated throughout the modelling process and be reflected in the resulting LCIA where error bars or probabilistic comparison methods like discernibility can allow transparent interpretation (Mendoza Beltran et al., 2018; Wender et al., 2014b). Spreading further along the uncertainty continuum, more qualitative aspects of uncertainty are reached as discussed in previous sections. Application of deliberative and participatory social science approaches helps to open-up discussion surrounding these uncertainties, encouraging reflection on the limits of knowledge and increasing awareness of other stakeholder perspectives (Ribeiro et al., 2016).

At early stages of technological development there are likely to be many sustainability aspects that cannot be fully evaluated due to high uncertainty, lack of knowledge or data, no available methods or simply lack of appropriate skills or resources. It is crucial, that while these “unmeasurables” are not empirically evaluated, that they are not discarded and that these unknowns are recorded and propagated to the interpretation stage for further deliberation.

### 6.3 Step 3: Interpretation and informed decision-making

#### 6.3.1 Consolidating and presenting the results

The interpretation stage is arguably the most important stage of CSA. How the results of assessments are consolidated and presented to stakeholders represents a key bottleneck where the risk of closing-down is high (Fig. 2). While aggregation and weighting involves subjective judgements and discards complexity regarding trade-offs and uncertainty, it is also challenging for non-technical stakeholders to understand the meaning and significance of raw sustainability assessment results, and thus there is a tension between understandability and robustness (Peace et al., 2017). Indeed, where life-cycle methods are already utilised to inform decision-making there are fears that the inherent subjectivities and uncertainties embedded within these methods may not be properly understood or reflected in the decisions taken by stakeholders who desire crisp answers to fuzzy sustainability questions (Sonnemann et al., 2018). Furthermore, even if the limitations, uncertainties and qualitative results of the assessment are presented, quantitative results presented in graphs, tables and diagrams will be easier and faster for decision-makers to understand and interpret with the danger that they are unfairly prioritised in decision-making.

With CSA we recommend taking a pragmatic approach. A certain degree of aggregation to more understandable “mid-points” or “end-points” may be appropriate, although the way in which this is carried out and the value-judgements involved must be made explicit. Where uncertainty levels are extremely high, one option is to focus on using analysis results for hotspot identification rather than articulating results in absolute terms. Employment of innovative presentation techniques, for example, the use of practical hands-on activities or diagrammatic presentation approaches can also help to alleviate these issues.

#### 6.3.2 Deliberation

More fundamentally, the challenge is not just in how the results are presented, but in how the empirical results are used and mobilised. CSA is not intended to give fixed answers to sustainability questions. Rather, it explores a set of possibilities and potential impacts (Olsen et al., 2018). To achieve this, we advocate a deliberative interpretation approach more appropriate for the inherent uncertainty and subjectivity of the sustainability concept. While this makes the process more complicated and does not result in crisp results, it reflects the true nature of the outputs and allows the propagation of uncertainty directly to decision-makers. Using deliberative activities like workshops to re-engage stakeholders allows empirical results gathered in the evaluation phase to be related to the formulation stage that they engaged with. During these deliberative activities the following aspects should be reflected upon:

• The meaning and significance of the results, including any unexpected results or significant hotspots. While the CSA practitioner will need to explain the results and ensure that stakeholders are able to make judgements based on an informed understanding of how the results were generated, the stakeholders themselves should be encouraged to derive their own interpretations.
• CSA practitioners should be clear about, and encourage reflection upon, the limitations of the evaluation results and encourage discussion of what the results can tell us and what they cannot. This can lead to the identification of important gaps and unknown impacts which could be investigated in future CSA cycles.
• Discussion of how differing worldviews might impact the interpretation of the results, and how this might lead to other societal stakeholders coming to different conclusions, helping to encourage reflexivity (Stilgoe et al., 2013). If a stakeholder takes a “vulnerable nature” worldview it can render uncertain, early-stage sustainability assessments almost irrelevant (Asveld and Stemerding, 2016). This process can be aided by the three different archetypes (hierarchist, individualist, egalitarian) used in the ReCiPe impact assessment methodology (Hofstetter et al., 2000, Huijbregts et al., 2017).
• To encourage responsiveness, the stakeholder participants should be asked to identify potential recommendations or actions, either to be taken now or responses which may become appropriate in the future, should possible but uncertain outcomes become clearer. This step provides opportunities to initiate value-sensitive design (VSD) (Friedman, 1996).

#### 6.3.3 An open and continuous process

During the process of CSA, there are various stages at which narrow framing and closing-down may occur prematurely (Fig. 2). To address this, the interpretation stage represents an opportunity to re-open the process by considering ambiguities, uncertainties, and alternative interpretations. The interpretation stage should not in any sense be considered an end-point. The process should remain open and continuous in order to be responsive to new information and developments, supporting an incremental approach (Lindblom, 1959). By setting out recommendations for further evaluation, the interpretation stage can act as the formulation stage for future rounds of CSA. This helps to ensure continuous evaluation and deliberation, feeding information into governance and decision-making as soon as it becomes available.

## 7 Options for operationalisation

So far, we have put forward a primarily conceptual exposition of CSA. Future research will necessitate the operationalisation of the framework in practice to enable further elaboration and refinement. CSA is designed to be continuous and iterative and therefore implicitly it must be applicable at a variety of stages of development and in different institutional contexts. While CSA would ideally be operationalised as early as possible in the development of emerging technologies, it could also be introduced to already well-developed technologies where it can help to re-open the governance process. Furthermore, where an LCA has already been undertaken a CSA framework could be introduced at the interpretation stage rather than the formulation stage to open-up discussion and explore options for further evaluation.

**Fig. 3:**
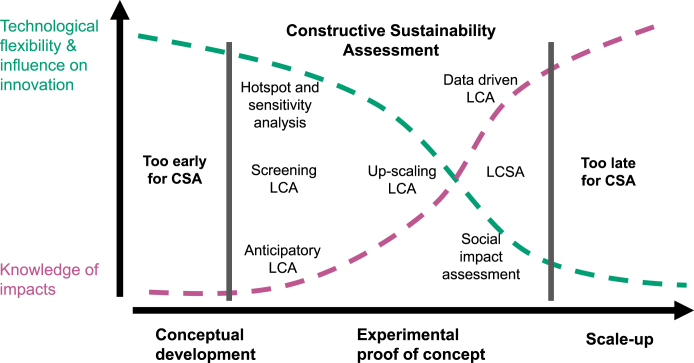
The Collingridge dilemma of social control for emerging technologies with options for CSA operationalisation overlaid.

In Fig. 3 we provide some suggested avenues and stages for operationalisation overlaid on our graphical elaboration of the Collingridge dilemma. During initial stages of emergence foresight, horizon scanning and public dialogues are already established, and CSA does not seek to replace these. Similarly, when a technology is well-developed and diffused the opportunities for CSA to have impact are minimal. The areas where CSA application is most pertinent is between these two stages, bridging the well-known “valley of death”. This is where the crucial design decisions are taken and experimentation occurs so is where knowledge of sustainability implications can be most pertinent.

Advanced emerging technologies are typically initiated in research environments. Operationalising a CSA process at this early stage will maximise the potential to influence research trajectories before lock-in becomes apparent. Furthermore, the trend of embedding RRI and related frameworks within natural science research programmes provides an entry point for CSA activities to being carried out (Karinen and Guston, 2010, Owen, 2014). Scientists should be engaged in the formulation process to inform the evaluation stage with subsequent deliberative interpretation workshops allowing the exploration of anticipated sustainability implications informed by available data. This provides opportunities for sustainability considerations to be integrated into emerging technologies at an early stage. Inviting broader stakeholders (e.g. non-governmental organisations, industry figures, regulators, members of the public) to participate in formulation and interpretation workshops would help to broaden the sustainability perspectives considered and facilitate shared agenda-setting. CSA could thus provide operationalisation to RRI by enabling improved anticipation of impacts, the inclusion of different viewpoint, responsiveness to changes and reflexivity on the part of technological actors (Stilgoe et al., 2013).

Meanwhile, the scale-up and commercialisation of emerging technologies is typically driven by firms. Firms developing technologies that promise sustainability benefits often engage consultants to carry out analyses like LCAs to back up such claims and promises. Building on this already established interest in sustainability assessment, the engagement of firms in the formulation stage of CSA would enable their sustainability claims to be made explicit. These claims can subsequently be evaluated, providing firms with an opportunity to demonstrate a more substantive commitment to sustainability by proactively incorporating sustainability considerations into the design of their products through deliberative interpretation. This would represent an example of value-sensitive design (VSD) (Friedman, 1996).

Perhaps the most promising avenue for operationalisation is broadening out from the limited environments of research institutes or firms to implement CSA within communities. This would bring added challenges in structuring the process but would also reflect the fact that large-scale sustainability transitions will require sociotechnical shifts as well as technological fixes (Markard et al., 2012). Engaging a wider range of societal actors would help to situate the governance of emerging technologies towards sustainability within their broader context, linking technological developments with the overarching need for sustainable consumption as well as sustainable production.

## 8 Conclusions

This article began by outlining and critiquing current approaches to assessing and governing emerging technologies which could help to realise the sustainability benefits they promise. Analytical assessment methods such as LCA represent well-established and powerful tools for the evaluation of sustainability promises, systematically probing assumptions and frequently revealing unexpected results. In recent years substantial progress has been made in reshaping these tools to grapple with new and ambitious demands such as the need for a more anticipatory viewpoint and to consider broader notions of sustainability. However, they still fail to grapple with many of the normative dimensions of sustainability and the uncertainties of emerging technologies. LCAs are too frequently employed in isolation to answer uncertain and complex sustainability questions which they are ill-equipped to answer. We believe the power of analytical approaches like LCA is maximised when they are grounded within a broader, more deliberative framework.

We have also explored more participatory and deliberative frameworks such as RRI which offer an alternative, more qualitative and reflective perspective. These approaches offer opportunities to open-up discussion and inform an incremental approach towards the sustainable development of emerging technologies, although they do not replicate the quantitative information that analytical sustainability assessments can generate. High-level frameworks for RRI exist, such as the UK EPSRC’s framework (Owen, 2014), but practical application has been lacking at low-TRLs. We argue that combining these frameworks, with a dedicated period of evidence and data collection using tools like LCA, can offer enhancements.

Thus, rather than simply comparing and contrasting deliberative and analytical approaches, we have developed a “third-way” in the form of the CSA framework, which emphasises their mutual complementarity. Analytical sustainability assessments are highly powerful tools for evaluating emerging technologies, however, when used in isolation they can be dangerous and instrumental. By grounding them within a deliberative and participatory approach the assumptions and ambiguities of analytical approaches can be explored and made explicit. Furthermore, we argue for the importance of context, with analytical methods enabling the exploration of the specific sustainability opportunities and implications relating to emerging technologies and their applications. Based on this, the balance of different methods and of participating stakeholders should intrinsically be linked to the context and specificities of the emerging technology in question.

The grand challenge of sustainability, perhaps the greatest challenge facing society, is highly complex. It involves problematic trade-offs that necessitate a systemic perspective. With emerging technologies, governance under high levels of uncertainty is required (Collingridge, 1980). To tackle this challenge requires the asking of complex questions to which there will not be easy answers. Therefore, rather than provide unrealistically clear solutions, CSA involves exploring options and rationalising uncertainties while reflecting on assumptions and alternative framings and perspectives. CSA emphasises the importance of maintaining an open discourse on emerging technologies while also engaging in the critical evaluation of promises and expectations. This maintains the two in productive and continuous tension in the search for incremental and constructive governance of emerging technologies. While CSA does not “solve” Collingridge’s dilemma, it actively tackles it through a continuously responsive process.

Through its four core design principles and three-step methodology, CSA provides a means to operationalise RRI for emerging technologies through the anticipatory and deliberative application of sustainability assessments. In doing so, CSA represents a means through which the emerging technologies can be governed in a continuous way from an early stage in order to realise the sustainability benefits they promise. Its capabilities and utility are maximised when applied early. Thus, resources and funding for assessments must be provided earlier, in parallel or ideally before significant funding is committed to the emerging technologies themselves resulting in the onset of path dependency and lock-in. Moreover, while CSA provides a route, it cannot provide the underlying incentives for sustainable development. In relation to emerging technologies, the promise of sustainability is frequently ambiguous, mobilised all too often for instrumental means. The widespread operationalisation of CSA and related frameworks would inform and enable real moves towards sustainability, although doing so will require substantive commitments to such processes by government, research organisations, industry, and non-governmental groups.

## Footnotes

1 “Cradle-to-gate” and “gate-to-gate” are also common variants with different system boundaries, chosen as appropriate for the system under study.
